# Supplementary material for: Comprehensive Genetic Analyses of Inherited Peripheral Neuropathies in Japan: Making Early Diagnosis Possible
Source: Biomedicines. 2022 Jun 29;10(7):1546. doi: 10.3390/biomedicines10071546 (PMC9312503; doi:10.3390/biomedicines10071546)
Supplement: Supplementary file 1 [file biomedicines-10-01546-s001.zip › biomedicines-1783315-supplementary.pdf]

Supporting information

Figure S1. RP-PCR of each repeat expansion genotypes in *RFC1* and MLPA of *MPZ* and *GJB1*. Multi-type bi-allelic *RFC1* repeat expansions, including [(AAGGG)exp/(AAGGG)exp] (patient 2), [(AAGGG)exp/(ACAGG)exp] (patient 8), [(ACAGG)exp/(ACAGG)exp] (patient 12), [(AAGGG)exp/(AAAGG)$_{13}$(AAGGG)exp] (patient 17), and [(ACAGG)exp/(AAAGG)$_{12}$(AAGGG)exp] (patient 18). MLPA analysis reveals a duplication of *MPZ* exon 2–6 (1 case) and a deletion of *GJB1* exon 2 (2 cases).

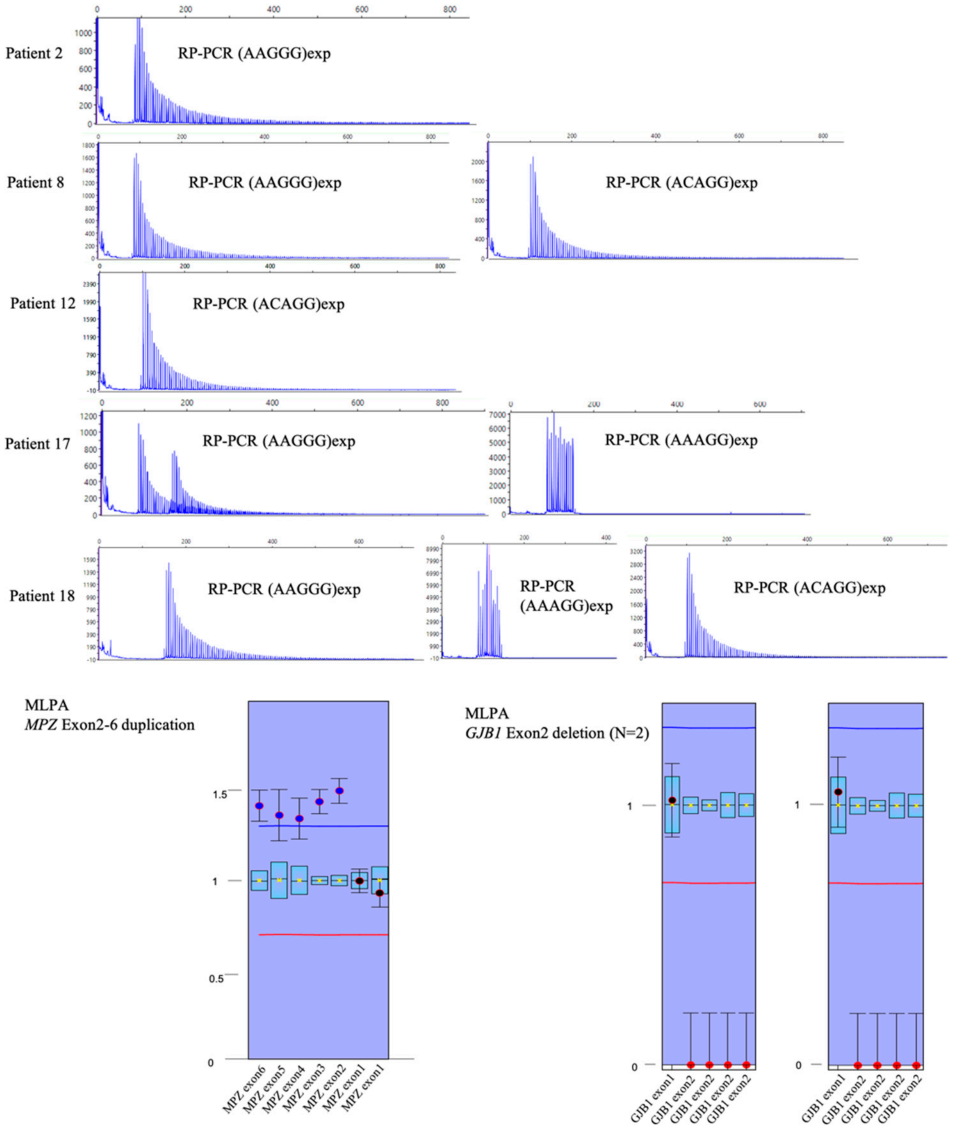

Table S1. Targeted IPN-related gene panels for DNA microarray and NGS.

Targeted 28 genes of Microarray Chip sequencing 2007-2012	*AARS1*	*APTX*	*DNM2*	*EGR2*	*GAN1*	*GARS*	*GDAP1*	*GJB1*
	HSPB1	*HSPB8*	*LITAF*	*LMNA*	*LMNA*	*MFN2*	*MPZ*	*MTMR2*
	NDRG1	*NEFL*	*PMP22*	*PRX*	*RAB7*	*SBF2*	*SETX*	*SH3TC2*
	SLC12A6	*SOX10*	*TDP1*	*YARS1*				
Targeted panel using Miseq (60 genes) 2012-2014	*AARS1*	*APTX*	*ARHGEF10*	*DHH*	*DNM2*	*EGR2*	*FGD4*	*FIG4*
	GAN	*GARS*	*GDAP1*	*GJB1*	*HARS*	*HK1*	*HOXD10*	*HSPB1*
	HSPB8	*KARS*	*LITAF*	*LMNA*	*MARS*	*MED25*	*MFN2*	*MPZ*
	MTMR2	*NDRG1*	*NEFL*	*PMP22*	*PRPS1*	*PRX*	*RAB7A*	*SBF2*
	SETX	*SH3TC2*	*SLC12A6*	*SOX10*	*TDP1*	*TRPV4*	*TTR*	*YARS*
	20 candidate gene							
Targeted panel using Ion Proton (72 genes) 2014-2021	*AARS*	*APTX*	*ARHGEF10*	*BSCL2*	*COA7*	*DCAF8*	*DCTN1*	*DHH*
	DHTKD1	*DNM2*	*DYNC1H1*	*EGR2*	*FBLN5*	*FBXO38*	*FGD4*	*FIG4*
	GALC	*GAN*	*GARS*	*GDAP1*	*GJB1*	*GJB3*	*GNB4*	*HARS*
	HK1	*HOXD10*	*HSPB1*	*HSPB3*	*HSPB8*	*IGHMBP2*	*INF2*	*KARS*
	KIF1A	*LITAF*	*LMNA*	*LRSAM1*	*MARS*	*MED25*	*MFN2*	*MME*
	MPZ	*MTMR2*	*NDRG1*	*NEFL*	*PDK3*	*PLEKHG5*	*PMP22*	*PRPS1*
	PRX	*RAB7A*	*REEP1*	*SACS*	*SBF1*	*SBF2*	*SETX*	*SH3TC2*
	SLC12A6	*SLC5A7*	*SOX10*	*SURF1*	*TDP1*	*TFG*	*TRIM2*	*TRPV4*
	TTR	*YARS*	*6 candidate gene*					
Targeted panel using Ion Proton (103 genes) 2022-	*AARS1*	*ABHD12*	*AIFM1*	*APTX*	*ARHGEF10*	*ATL1*	*ATL3*	*ATP1A1*
	BAG3	*BICD2*	*BSCL2*	*COA3*	*COA7*	*COX6A1*	*DCTN1*	*DHTKD1*
	DNAJB2	*DNM2*	*DNMT1*	*DRP2*	*DYNC1H1*	*EGR2*	*FBLN5*	*FGD4*
	FIG4	*FUS*	*GALC*	*GAN*	*GARS1*	*GDAP1*	*GJB1*	*GJB3*
	GNB4	*GNE*	*HARS1*	*HINT1*	*HOXD10*	*HSPB1*	*HSPB3*	*HSPB8*
	IGHMBP2	*INF2*	*KARS1*	*KIF1A*	*LAMA2*	*LITAF*	*LMNA*	*LRSAM1*
	MARS1	*MCM3AP*	*MFN2*	*MME*	*MORC2*	*MPV17*	*MPZ*	*MTMR2*
	NAGLU	*NDRG1*	*NEFH*	*NEFL*	*NTRK1*	*PDK3*	*PDXK*	*PLEKHG5*
	PMP2	*PMP22*	*PNKP*	*POLG*	*POLR3B*	*PRDM12*	*PRPS1*	*PRX*
	RAB7A	*REEP1*	*SACS*	*SBF1*	*SBF2*	*SCN11A*	*SCN9A*	*SCO2*
	SETX	*SH3TC2*	*SIGMAR1*	*SLC12A6*	*SLC25A46*	*SLC52A3*	*SLC5A7*	*SOD1*
	SORD	*SPG11*	*SPTLC1*	*SPTLC2*	*SURF1*	*TDP1*	*TFG*	*TRPV4*
	TTR	*VCP*	*WARS1*	*WNK1*	*YARS1*	*2 candidate gene*		

Table S2. Primers of flanking and repeat-primed PCR for *RFC*1 repeat expansion analyses

**Protocol**	**Primer (5'-3')**
Flanking PCR	Forward: TCAAGTGATACTCCAGCTACACCGTTGC
	Reverse: CAGCATTGTGGGAGACAGGCCAATCACT
Repeat-primed PCR	**AAGGG (pathogenic)**
	Forward: [6FAM]TCAAGTGATACTCCAGCTACACCGT
	Anchor: CAGGAAACAGCTATGACC
	Reverse: CAGGAAACAGCTATGACCGGGAAGGGAAGGGAAGGGAA
	**ACAGG (pathogenic)**
	Forward: [6FAM]TCAAGTGATACTCCAGCTACACCGT
	Anchor: CAGGAAACAGCTATGACC
	Reverse: CAGGAAACAGCTATGACCACAGGACAGGACAGGACAGG
	**AAAAG (benign)**
	Forward: [6FAM]TCAAGTGATACTCCAGCTACACCGT
	Anchor: CAGGAAACAGCTATGACC
	Reverse1: CAGGAAACAGCTATGACCAACAGAGCAAGACTCTGTTTCAAAAAAG AAAAGAAAAGAAAAGAAAA
	Reverse2: CAGGAAACAGCTATGACCAACAGAGCAAGACTCTGTTTCAAAAAGA AAAGAAAAGAAAAGAAAA
	Reverse3: CAGGAAACAGCTATGACCAACAGAGCAAGACTCTGTTTCAAAAGAA AAGAAAAGAAAAGAAAA
	**AAAGG (benign)**
	Forward: [6FAM]TCAAGTGATACTCCAGCTACACCGT
	Anchor: CAGGAAACAGCTATGACC
	Reverse: CAGGAAACAGCTATGACCGGAAAGGAAAGGAAAGGAAA

Table S3. Clinical and electrophysiological information of 18 cases with *RFC1* repeat expansion

Patient	Pt 1	Pt 2	Pt 3	Pt 4	Pt 5	Pt 6	Pt 7	Pt 8	Pt 9	Pt 10	Pt 11	Pt 12	Pt 13	Pt 14	Pt 15	Pt 16	Pt 17	Pt 18
RFC1 repeat	(AAGGG)exp						(AAGGG)exp				(ACAGG)exp						(AAGGG)exp	(ACAGG)exp
	(AAGGG)exp						(ACAGG)exp				(ACAGG)exp						(AAAGG)13<br>(AAGGG)exp	(AAAGG)12<br>(AAGGG)exp
Onset age	33	40	49	50	0	1	72	53	64	10	44	65	60	72	71	76	25	50
Exam age	37	43	53	70	1	40	78	70	76	16	45	74	72	74	75	80	45	65
Sex	M	M	M	M	M	M	M	M	M	M	M	M	M	F	M	F	M	F
Genotype	S	S	NA	S	S	S	S	S	R	S	S	S/R	S	S	S	S	S	S
Muscle weakness	+	+	+	+	+	+	+	+	+	+	+	+	+	+	+	−	+	+
Upper limb MMT (proximal)	5	5	3-4	5	NA	NA	4	5	5	4	5	5	5	5	5	5	5	5
Upper limb MMT (distal)	5	5	4-5	4	NA	NA	1	4	4	3	4	5	4	5	3	5	4	4
Lower limb MMT (proximal)	5	5	3-4	5	NA	3	4	5	5	4	5	5	4	4	5	5	5	5
Lower limb MMT (distal)	3	4	4-5	5	NA	4	0-1	4	4	3	5	4	4-	4	4	5	4	5
Muscle atrophy	+	+	NA	+	−	NA	+	−	NA	+	−	+	+	+	+	+	+	+
DTR	Decrease	Decrease	NA	Absent	Decrease	Decrease	Absent	Absent	Decrease	Hyper	Decrease	Absent	Absent	Absent	Absent	Absent	Normal	Absent
Sensory disturbance	+	+	+	+	NA	+	+	+	+	+	+	+	+	+	+	+	−	+
Cerebellar ataxia	−	−	+	−	NA	NA	−	+	−	−	−	+	−	−	+	−	−	+
Cerebellar atrophy	−	−	Mild atrophy	NA	NA	NA	−	−	NA	NA	NA	+	−	NA	+	NA	−	−
Vestibular dysfunction	−	−	NA	−	NA	NA	−	+	−	−	−	NA	−	−	NA	NA	NA	+
Choronic cough	−	NA	NA	NA	NA	NA	−	+	NA	−	−	NA	+	+	NA	NA	NA	NA
Pyramidal sign	−	−	NA	−	−	−	−	−	−	+	−	−	−	−	−	−	−	−
Parkinsonism	−	NA	+	−	−	NA	−	−	−	−	−	−	−	−	−	−	−	−
Cognitive impairment	−	NA	NA	−	NA	NA	−	−	NA	ADHD	−	NA	−	−	−	−	−	−
Involuntary movement	−	NA	NA	−	NA	NA	−	−	−	−	−	−	−	Tremor	−	−	−	−
Autonimic dysfunction	−	NA	NB, OH, ED	−	NA	NA	−	Constipation Hypohidrosis	Constipation Pollakiuria	−	−	−	−	−	−	OH, NB	hypohidrosis	−
Muscle cramp	+	NA	NA	NA	NA	NA	−	+	NA	−	−	Fasciculation	−	+	NA	NA	NA	−
Creatine Kinase (IU/dL)	450	NA	400-2200	NA	NA	339-700	322	−	NA	27	−	91	385	125	531	NA	386	85
Other symptom	−	−	−	−	−	−	−	Epilepsy	Diplopia	Neurofibromatosis	−	−	−	−	−	−	−	−
Median MNCV (m/s)	57.1/57.9	59.2/56.5	NA	55.4	37.4	56	46	49	48	46.8	57	53.3	43.7	56.4	57.5	Normal	50.6	56.5
Median CMAP (mV)	9.94/10.5	14.7/16.4	NA	5.3	7.4	11.7	0.3	8.6	4.52	NA	9.5	5.5	8.7	3.4	4.13	Normal	6.16	8.48
Median SCV (m/s)	30.2	54.5/58.8	NA	NE	46.4	NA	NE	NE	31	NA	39.8	NE	NE	NE	NE	Decrease	48	58.0
Median SNAP (μV)	64	21.4/28.7	NA	NE	5.1	NA	NE	NE	2	NA	12.8	NE	NE	NE	NE	Decrease	0.4	2.6
Tibial MNCV (m/s)	43.8/42.3	47.9/43.6	NA	41.4	45.5	43	39.5	34	38	38.6	47.9	38.0	35.8	42.1	31.1	Decrease	40.2	43.8
Tibial CMAP (mV)	10.2/6.95	20.1/6.9	NA	2.4	1	7	0.2	1.5	5.8	NA	8.6	1.2	4.1	9.5	0.49	Decrease	3.2	6.15
Sural SCV (m/s)	58.8/52.9	47.8/49.2	NA	NE	43.1	NA	NE	NE	NE	NA	NE	NE	NE	NE	NE	NE	NE	NE
Sural SNAP (μV)	7.6/9.3	12.9/9.4	NA	NE	11	NA	NE	NE	NE	NA	NE	NE	NE	NE	NE	NE	NE	NE

M: male; F: female; S: sporadic; R: autosomal recessive; R/S: sporadic or autosomal recessive; MMT: manual muscle test; NA: not available; DTR: deep tendon reflex; ADHD: attention-deficit hyperactivity disorder; NB: neurogenic bladder; OH: orthostatic hypotension; ED: erectile dysfunction; CK: creatine kinase (U/L); MNCV: motor nerve conduction velocity; CMAP: compound motor action potential; SCV: sensory nerve conduction velocity; SNAP: sensory nerve action potential; NE: not evoked. Normal range: median CMAP > 3.1 mV; median MCV > 49.6 m/s; median SNAP > 7.0 μV; median SCV > 47.2 m/s; tibial CMAP > 4.4mV; tibial MCV > 41.7 m/s; sural SNAP > 5.0 μV; sural SCV > 40.8 m/s; CK (male) 59–248 IU/mL and (female) 41–153 IU/mL.
